# *Linc-GALMD1* Regulates Viral Gene Expression in the Chicken

**DOI:** 10.3389/fgene.2019.01122

**Published:** 2019-11-14

**Authors:** Yanghua He, Bo Han, Yi Ding, Huanmin Zhang, Shuang Chang, Li Zhang, Chunfang Zhao, Ning Yang, Jiuzhou Song

**Affiliations:** ^1^Department of Human Nutrition, Food and Animal Sciences, University of Hawaii at Manoa, Honolulu, HI, United States,; ^2^Department of Animal and Avian Sciences, University of Maryland, College Park, MD, United States; ^3^National Engineering Laboratory for Animal Breeding, Department of Animal Genetics and Breeding, College of Animal Science and Technology, China Agricultural University, Beijing, China; ^4^Avian Disease and Oncology Laboratory, Agricultural Research Service, USDA, East Lansing, MI, United States; ^5^College of Veterinary Medicine, Shandong Agricultural University, Tai’an, China; ^6^Institute of Animal Science and Veterinary Medicine, Beijing Academy of Agriculture and Forestry Sciences, Beijing, China

**Keywords:** *Linc-GALMD1*, long intergenic non-coding ribonucleic acids, Marek’s disease, Marek’s disease virus, Chicken, *Meq* gene

## Abstract

A rapidly increasing number of reports on dysregulated long intergenic non-coding RNA (lincRNA) expression across numerous types of cancers indicates that aberrant lincRNA expression may be a major contributor to tumorigenesis. Marek’s disease (MD) is a T cell lymphoma of chickens induced by Marek’s disease virus (MDV). Although we have investigated the roles of lincRNAs in bursa tissue of MDV-infected chickens in previous studies, the molecular mechanisms of lincRNA functions in T cells remain poorly understood. In the present study, *Linc-GALMD1* was identified from CD4+ T cells and MSB1 cells, and its expression was significantly downregulated in MD-resistant line of birds in response to MDV challenge. Furthermore, loss-of-function experiments indicated that *linc-GALMD1* significantly affected the expression of 290 genes *in trans*. Through integrated analysis of differentially expressed genes (DEGs) induced by MDV and *linc-GALMD1*, we found that *IGLL1* gene expression levels had a positive correlation with the degree of MD infection and could potentially serve as an indicator for clinical diagnosis of MD. Moreover, an interaction between MDV and *linc-GALMD1* was also observed. Accordingly, chicken embryonic fibroblast cells were inoculated with MDV with and without the *linc-GALMD1* knockdown, and the data showed that *linc-GALMD1* could repress MDV gene expression during the course of MDV infection. These findings uncovered a role of *linc-GALMD1* as a viral gene regulator and suggested a function of *linc-GALMD1* contributing to tumor suppression by coordinating expression of MDV genes and tumor-related genes and regulating immune responses to MDV infection.

## Introduction

Marek’s disease (MD) is a naturally occurring rapid-onset aggressive T-cell lymphoma of poultry, and it is caused by Marek’s disease virus type 1 (MDV-1). In chickens, virulent MDV undergoes four overlapping infection stages, having critical consequences that contribute to viral persistence and pathogenesis in the host: early cytolytic, latent, late cytolytic, and transformation (Biggs, 1968). In the early cytolytic stage, the virus replicates in macrophages, B and T lymphocytes, and the MDV genome can be detected in B and T lymphocytes as early as 2 days post-infection (dpi). Following the lytic phase of infection, latent (non-replicating) infection occurs primarily in CD4+ T cells (T-helper cells) that are capable of being transformed around 7 dpi. The MDV genome is highly transcribed during this latent infection, although no viral or tumor antigens are expressed, and the expression of Marek’s EcoRI-Q-encoded protein (*Meq*) transcripts is similar to that of MDV-transformed cells (Calnek et al., 1984; Arumugaswami et al., 2009). In the late cytolytic stage from 14 to 21 dpi, latently infected cells carry the virus to the thymus, bursa, and some epithelial tissues. Necrosis of lymphocytes and epithelial cells is accompanied by pronounced inflammation, infiltration of mononuclear cells and heterophils, and (for the bursa and thymus) severe atrophy (Baigent and Davison, 2004).

Long non-coding RNAs (lncRNAs) are non-coding transcripts longer than 200 nucleotides. lncRNAs can regulate gene expression by different mechanisms including lncRNA transcription-dependent regulation of neighbor genes, binding onto DNA/-RNA binding proteins to control their cellular localization or transmit information among chromosomes, supporting nuclear structures or chromatin-modifying complexes, and acting as sponges of microRNAs (Bhat et al., 2016; Marchese et al., 2017). Long intergenic non-coding RNAs (lincRNAs) as a type of lncRNAs were indicated to play key roles in cancer-related gene regulatory systems by establishing chromatin domains in an allele- and cell type-specific manner, and the disorder of their expression is thought to promote cancer cell proliferation, invasion, and metastasis (Tsai et al., 2011; Bhat et al., 2016). In addition, transcriptional profiling has been proved to be a powerful tool for discovering lncRNAs with biological functions (Lin et al., 2014). Our previous studies have investigated the signatures of lincRNAs in bursa tissue of chickens with MD, and a candidate lincRNA, *linc-satb1*, was found to play a crucial role in MD immune response by regulating a nearby protein-coding gene *SATB1 in cis* (He et al., 2015). Nevertheless, MD as a T cell lymphoma is still largely unexplored concerning the biological functions of lincRNAs in T cells. The MSB1 lymphoblastoid cell line is an MDV-transformed CD4+ T-cell line derived from a spleen lymphoma induced by the BC-1 strain of MDV-1 (Akiyama and Kato, 1974; Hirai et al., 1990) (Yao et al., 2008). The MSB1 cell line has a CD4+ phenotype while it has both integrated and circular copies of the MDV-1 genome, which shares many properties of MD tumors. Thus, the MSB1 cell line is a commercial and unique cell-model that is closer to the latently infected CD4+ T-lymphocyte. Consequently, in the present work, we identified lincRNAs in CD4+ T cells isolated from MDV-infected chickens at the late cytolytic stage. A differentially expressed lincRNA, *linc-GALMD1*, between infected and non-infected chickens was focused on to explore the biological functions and molecular mechanism of *linc-GALMD1* in MD. Loss-of-function experiments of *linc-GALMD1* and the following transcriptomic sequencing in MSB1 cells were performed to explore its roles in MD-related gene regulatory systems. Furthermore, MDV-infection assays were further conducted in chicken embryonic fibroblast (CEF) cells with the *linc-GALMD1* knockdown. Our results suggested that *linc-GALMD1* may represent a novel indicator of poor prognosis and may serve for the diagnosis and treatment of MD. This study further advances our understanding of the sophisticated regulation of lincRNAs such as *linc-GALMD1* as a viral regulator of MD pathogenesis.

## Results

### Long Intergenic Non-Coding Ribonucleic Acids Identification in Marek’s Disease Chickens

To explore changes of protein-coding genes and non-coding transcripts, as well as their interactions upon MDV infection in chickens, transcriptomic sequencing was conducted in eight RNA samples extracted from chicken CD4+ T cells isolated from individuals of F_0_ generation before and after Marek’s disease viral infection. F0 generation chickens include two highly inbred parental lines 6_3_ and 7_2_. All chicken lines were maintained at the Avian Disease and Oncology Laboratory (ADOL) (Bacon et al., 2000).

A total of 274 candidate lincRNAs were identified from chickens of the two parental lines 6_3_ and 7_2_, using the data analysis pipeline as previously described (He et al., 2015). Differentially expressed lincRNAs were captured between infected and non-infected chickens by a p-value less than 0.2 (**Table 1** and **Supplementary Sheet 1**). The 16 differentially expressed lincRNAs were selected to confirm their expression in CD4+ T cells by qPCR, and results for 94% of lincRNAs analyzed were consistent with RNA-seq results (**Figure S1**).

**Table 1:** The statistics of candidate long intergenic non-coding RNAs (lincRNAs) and differentially expressed lincRNAs in lines 6_3_ and 7_2_ (p-value < = 0.2).

Groups	Number	
Total lincRNAs	274	
Differentially expressed lincRNAs	6_3_ I *vs.* 6_3_ N	41
	7_2_ I *vs.* 7_2_ N	53
	6_3_ I *vs.* 7_2_ I	94
	6_3_ N *vs.* 7_2_ N	56

### Loss of Function of *linc-GALMD1* in MSB1 Cells

**Figure 1:**
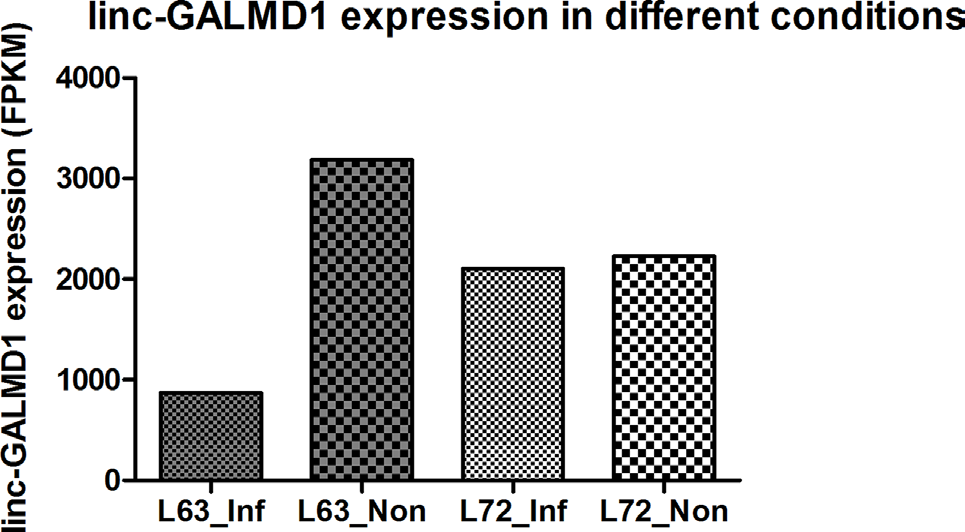
*linc-GALMD1* expression in CD4+ T cells with different conditions in two chicken lines.

**Figure 2:**
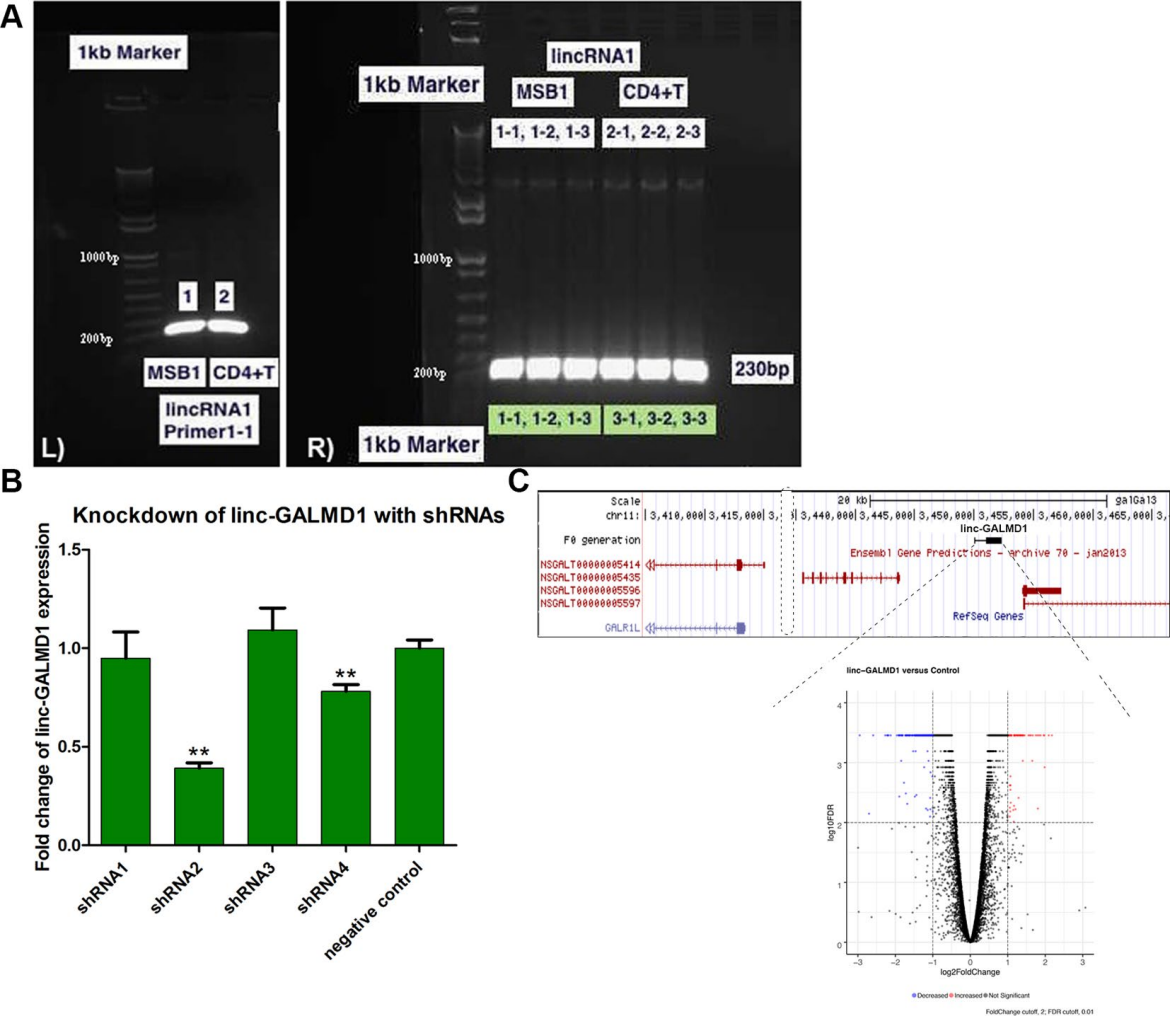
Structure confirmation of *linc-GALMD1* in CD4+ T cells and MSB1 cells. The primer pairs were designed to span over two transcripts of *linc-GALMD1*. **(A)** *linc-GALMD1* confirmation with double-stranded cDNA (dscDNA) from CD4+ T cells and MSB1 cells (L panel) and with plasmid DNA from multiple colonies of CD4+ T cells and MSB1 cells, respectively (R panel). **(B)** Knockdown efficiency of *linc-GALMD1* with four short hairpin RNAs plus a negative control. **(C)** The *linc-GALMD1* knockdown. Top: genomic locus containing the *linc-GALMD1*. Bottom: volcano plot of 290 differentially expressed genes affected by knockdown of the *linc-GALMD1* (|log2fold change| ≥ 1 and FDR ≤ 0.01). Red dots represent genes with increased expression after the linc-GALMD1 knockdown and blue dots mean genes with decreased expression, gray dots indicate genes with non-significant expression change after the long intergenic non-coding RNA knockdown. **The structure of linc-GALMD1 was detected in CD4+ T cells and MSB1 cells since we identified the lincRNA from CD4+ T cells and used MSB1 cells for loss-of-function assays of the lincRNA, which confirmed that our functional validation system works and linc-GALMD1 does relate to Marek’s Disease Virus infection.

*Linc-GALMD1* was found to be significantly differentially expressed between infected and non-infected chickens for MD resistant line 6_3_, but no expression difference was found for susceptible line 7_2_ (**Figure 1**). To explore possible roles for *linc-GALMD1* in chicken Marek’s disease, loss-of-function experiments of *linc-GALMD1* were performed in the MSB1 cell line that is a commercial MDV-transformed CD4+ T-cell line derived from lymphomas of chickens with MD. Primarily, the structure of *linc-GALMD1* was detected in CD4+ T cells and MSB1 cells by ordinary PCR with the primer pairs designed spanning over two exons of *linc-GALMD1*. PCR cloning and sequencing were conducted and further indicated that *linc-GALMD1* exists in both CD4+ T cells and MSB1 cells (**Figure 2A**).

To perform loss-of-function experiments of *linc-GALMD1*, we generated five lentiviral-based short hairpin RNAs (shRNAs) targeting *linc-GALMD1*, including one negative control shRNA in MSB1 cells. **Figure 2B** shows that shRNA2 and shRNA4 successfully targeted *linc-GALMD1* and significantly reduced its expression compared to negative control in MSB1 cells (p-value < 0.01, *t*-test). The best shRNA, shRNA2, was selected for final knockdown experiments and further RNA sequencing in MSB1 cells.

### Linc-GALMD1 Affects Gene Expression *In Trans*

**Figure 3:**
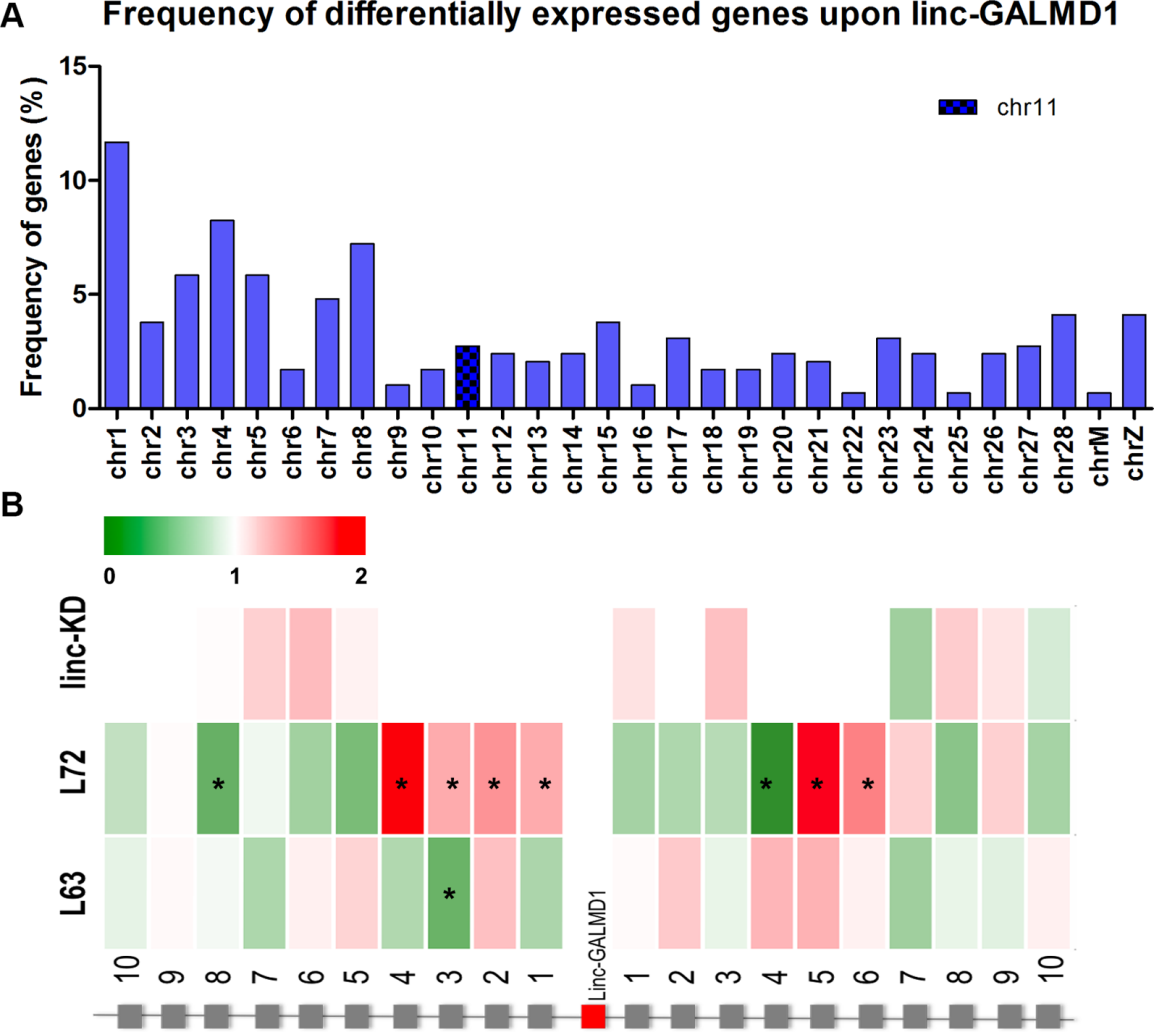
*linc-GALMD1* knockdown effects on gene expression. **(A)** The frequency of 290 differentially expressed genes affected by *linc-GALMD1* on chromosomes. **(B)** Effects of knockdown of *linc-GALMD1* on 10 neighboring genes on each side. Expression changes after Marek’s disease virus infection in chicken lines 6_3_ and 7_2_ are shown through these 10 neighboring genes on each side (green: down-regulation; red: up-regulation; star means genes with |log2fold change| ≥ 1).

Following the *linc-GALMD1* knockdown in MSB1 cells, RNA sequencing results demonstrated that *linc-GALMD1* significantly affected expression of 290 genes (|log2fold change| ≥ 1 and FDR ≤ 0.01) (**Figure 2C**), which are distributed on all chromosomes with only 3% of differentially expressed genes located on chromosome 11 that contains *linc-GALMD1* (**Figure 3A**). This suggests that *linc-GALMD1* is more likely to affect gene expression *in trans*. To examine whether *linc-GALMD1* could affect gene expression *in cis*, 10 neighboring genes upstream and another 10 downstream of *linc-GALMD1* were analyzed to determine their expression change upon the *linc-GALMD1* knockdown and MDV infection, respectively (**Figure 3B**). The results indicated that very few genes were up-regulated after the *linc-GALMD1* knockdown. However, their expression was significantly changed after MDV infection in line 6_3_ and 7_2_. Also, some genes had distinct reactions to MDV infection in resistant line 6_3_ and susceptible line 7_2_. Consequently, we hold the view that the effects of MDV infection on these 20 neighboring genes are far greater than the effects of *linc-GALMD1*.

In our previous study, we identified enhancers in MSB1 cells by deoxyribonuclease sequencing (He et al., 2014). An enhancer at 243 kb upstream of *linc-GALMD1* and another one at 2 Mb downstream were captured (**Figure S2**). Furthermore, the conservation of *linc-GALMD1* and neighboring enhancers was investigated. *Linc-GALMD1* and the upstream enhancer were not found in the human and mouse, but the downstream enhancer, next to *AKTIP* gene, was found across the chicken, human, and mouse, which provided some clues for decoding the chicken genome to explore regulatory elements involved in molecular mechanisms of MD.

### Protein-Coding Gene Changes Induced by *linc-GALMD1* and Marek’s Disease Virus

**Figure 4:**
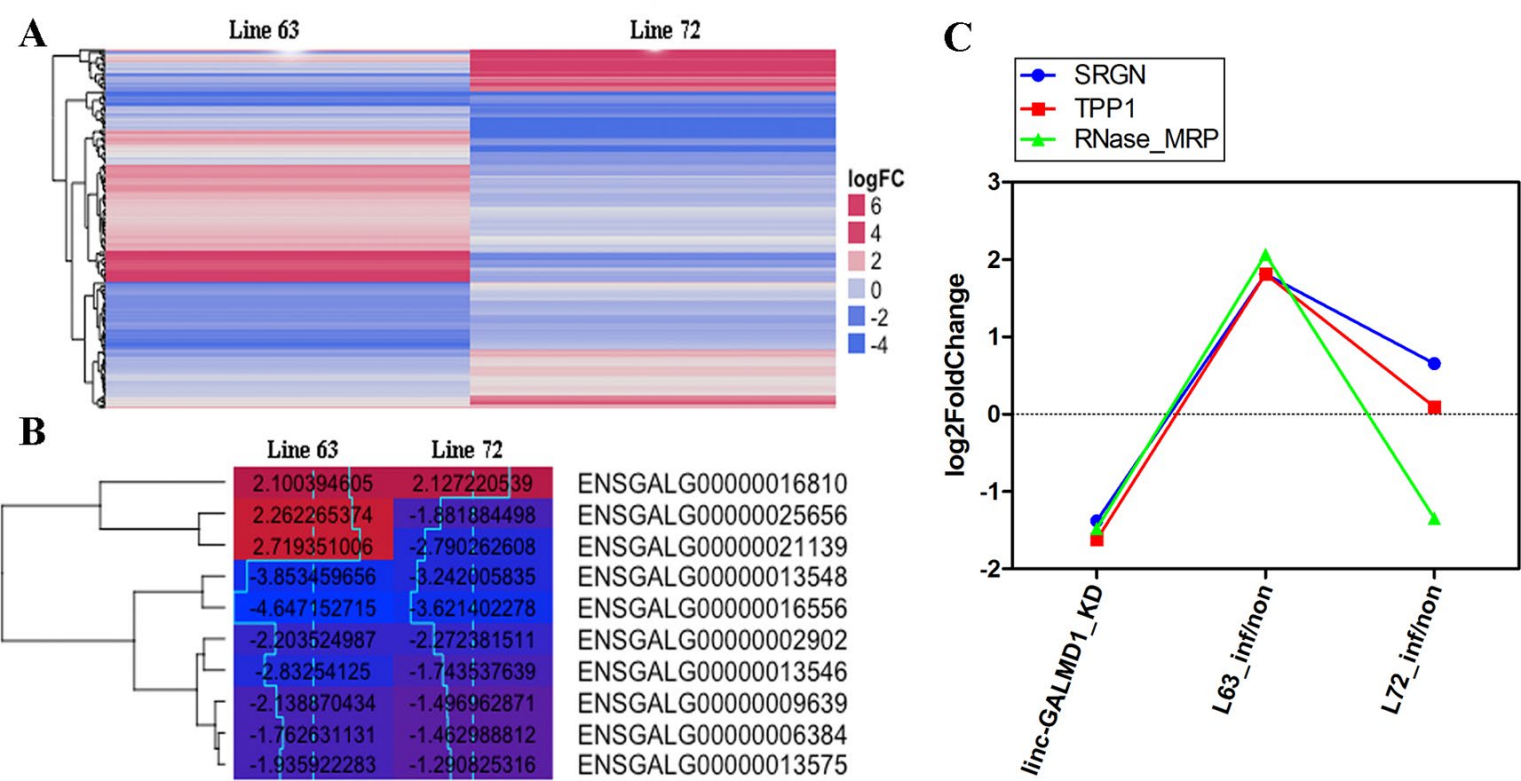
Gene expression changes induced by Marek’s disease virus (MDV) and *linc-GALMD1*. **(A)** The heatmap of differentially expressed genes in CD4+ T cells between infected and non-infected chickens in line 6_3_ or 7_2_ (|log2fold change| ≥ 1 and FDR ≤ 0.1). Red: up-regulation in non-infected chickens against infected chickens, blue: down-regulation in non-infected chickens. **(B)** The heatmap of 10 differentially expressed genes in CD4+ T cells between infected and non-infected chickens in both lines 6_3_ and 7_2_ (|log2fold change| ≥ 1 and FDR ≤ 0.1). The numbers labeled on each box are log2-fold-changes for the corresponding gene in a specific condition. Red: higher expression in non-infected chickens compared to infected chickens, blue: lower expression in non-infected chickens compared to infected chickens. **(C)** Log2-fold-changes of the differentially expressed genes (DEGs) before and after *linc-GALMD1* was knocked down in MSB1 cells, and that of DEGs in CD4+ T cells between infected chickens of line 6_3_ by MDV and non-infected chickens as well. (SRGN: ENSGALG00000004167; TPP1: ENSGALG00000022706; RNase_MRP: ENSGALG00000025557).

To further characterize differentially expressed genes (DEGs) induced by MDV and *linc-GALMD1*, respectively, differential expression of genes was analyzed in CD4+ T cells between MDV-infected and non-infected chickens in line 6_3_ and 7_2_, and before and after the *linc-GALMD1* knockdown in MSB1 cells as well. A total of 116 DEGs were found between infected- and non-infected chickens for line 6_3_, in which 71 genes were expressed higher in non-infected chickens than infected, namely, up-regulated genes; and the remaining 45 genes were down-regulated after MDV infection. For line 7_2_, 105 DEGs were found, and 68 genes were up-regulated and 37 were down-regulated after MDV infection (|log2fold change| ≥ 1 and FDR ≤ 0.1, **Figure 4A** and **Figure S3**). Gene Ontology (GO) analysis of these genes demonstrated that most genes are involved in nucleic acid binding and nucleotide-binding processes. In addition, a portion of genes participates in immune activities. Interestingly, most down-regulated genes that were highly expressed in infected chickens actively participate in viral reproduction, immune response, cell proliferation, cell killing, and cell death compared to up-regulated genes for both of line 6_3_ and 7_2_, which indicates that the immune systems of infected chickens were activated to defeat MDV infection (**Figure S4**). Ten genes were differentially expressed in both lines 6_3_ and 7_2_ (**Figure 4B**), in which two genes had distinct expression directions in the two chicken lines, seven genes were down-regulated, and one gene was up-regulated for both lines. These two line-specific genes are *IGLL1* (ENSGALG00000021139) and 5.8S rRNA (ENSGALG00000025656). *IGLL1* gene was lowly expressed in infected chickens for resistant line 6_3_, but it was highly expressed in infected chickens for susceptible line 7_2_, which implies that *IGLL1* could be a susceptible gene or a marker for clinical diagnosis of MD.

**Figure 5:**
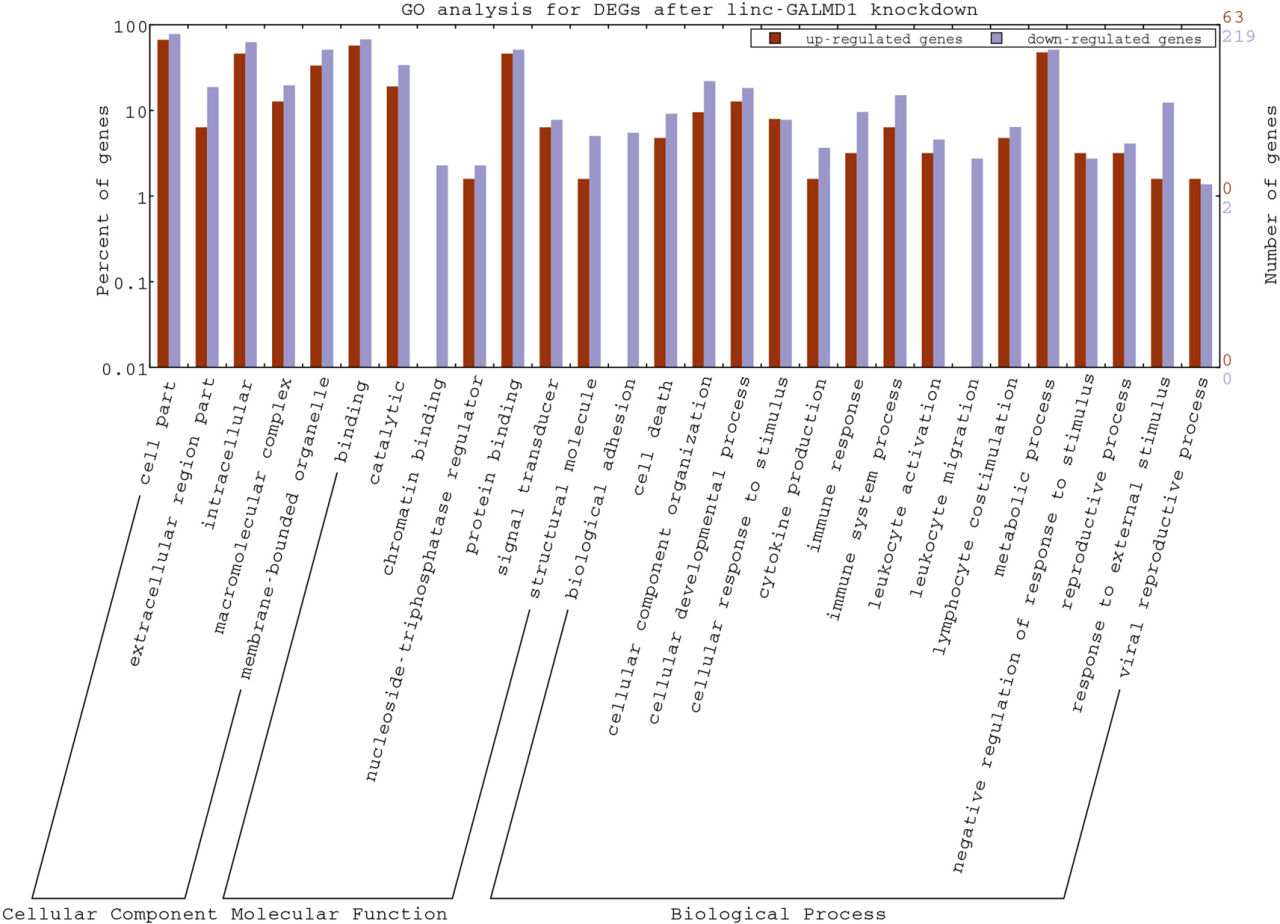
Gene Ontology Annotation Plotting of differentially expressed genes (DEGs) after the *linc-GALMD1* knockdown by WEGO. The BGI WEGO (Web Gene Ontology Annotation Plotting) was used to functionally categorize differentially expressed genes by cell component, molecular function, and biological process based on the chicken GO annotation information from Ensembl-BioMart database. Gene numbers and percentages (on a log scale) are listed for each category.

As mentioned above, the *linc-GALMD1* knockdown significantly affected the expression of 290 genes (**Figure 2C**), in which 64 genes were up-regulated and 226 genes were down-regulated after the *linc-GALMD1* knockdown. By GO analysis of these DEGs, we found that these genes were very active in extracellular and intracellular regions and they participate in macromolecular complex binding and transmembrane transporter activities, which hinted that this lincRNA could play roles in the cell nucleus, cell cytoplasm, and intercellular regions to support transporter activities. It is worth noting that down-regulated genes are preferentially involved more in immune response, cell motion, cell death, leukocyte activation and migration, as well as lymphocyte costimulation than up-regulated genes (**Figure 5**), which suggested that the immune system became less active after the *linc-GALMD1* knockdown in MSB1 cells, which is different from responses of the immune system to MDV infection that is the immune system may be activated when a chicken is infected by MDV.

Based on integrated analysis of RNA sequencing upon MDV infection and upon the *linc-GALMD1* knockdown, only three genes were found to be differentially expressed after *linc-GALMD1* knockdown in MSB1 cells, and after MDV infection in CD4+T cells for line 6_3_ (**Figure 4C**). The results indicated that their expression was significantly decreased after the *linc-GALMD1* knockdown. In addition, their expression was lower in infected chickens compared to non-infected chickens for resistant line 6_3_, but no significant expression difference was found for line 7_2_, which further illustrated that *linc-GALMD1* could play roles during MDV infection for chickens.

### Interactions Between *linc-GALMD1* and Marek’s Disease Virus

**Figure 6:**
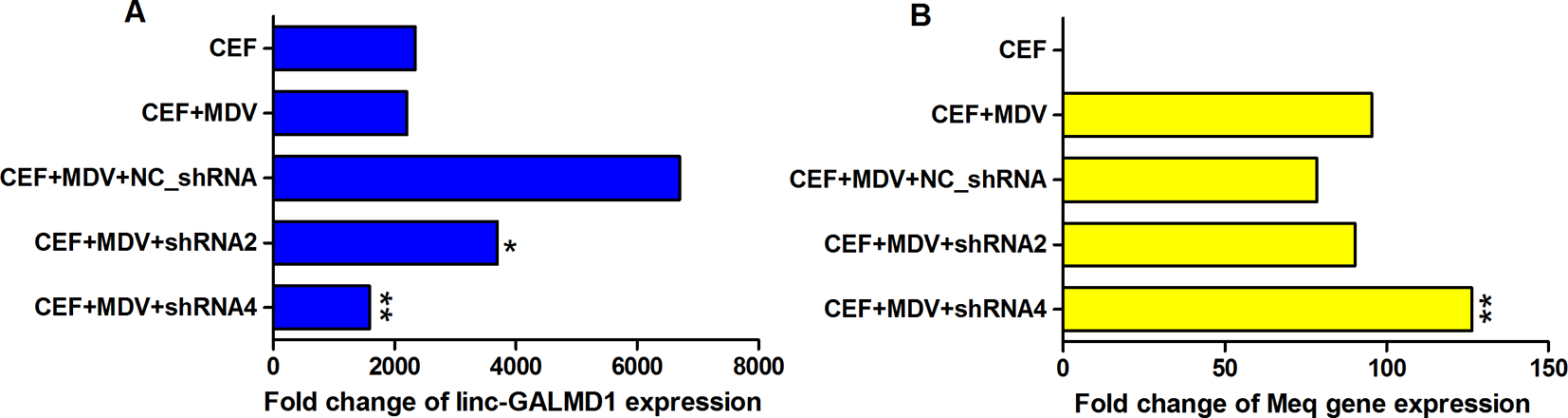
The interaction between *linc-GALMD1* and Marek’s disease (MD) virus in chicken embryonic fibroblast (CEF) cells. CEF cells were infected by MDV and Marek’s EcoRI-Q-encoded protein (*Meq*) gene represents MDV virulence. The change of MDV loads detected by copy numbers of the *Meq* gene was measured by quantitative PCR assay after the *linc-GALMD1* knockdown by shRNA2 and shRNA4. **(A)** The housekeeping gene, *GAPDH*, was selected as a control to calculate the relative expression of *linc-GALMD1* expression for all conditions. **(B)** For *Meq* gene, PCCA gene was used as a negative control. One asterisk represents p-value ≤ 0.05, two asterisks represent p-value ≤ 0.01, *t*-test.

To determine whether MDV could interact with *linc-GALMD1*, MDV infection was performed in CEF cells with and without *linc-GALMD1* knockdown by shRNA2 and shRNA4, respectively. It was known that MDV induces rapid-onset T-cell lymphomas in chickens and that T-cell transformation requires the expression of a viral protein called *Meq*, whose changes in the coding sequence of *Meq* correlated with increased virulence (Kumar et al., 2012). **Figure 6** demonstrates that *linc-GALMD1* was significantly knocked down by shRNA2 and shRNA4 compared to a negative control shRNA upon MDV infection (**Figure 6A**). Meanwhile, *Meq* copy numbers increased after the *linc-GALMD1* knockdown compared to the condition with MDV infection and a negative control shRNA. *Meq* copy numbers increased more when *linc-GALMD1* was knocked down more by shRNA4 than by shRNA2 (p-value ≤ 0.01, **Figure 6B**). These results indicate that *linc-GALMD1* could repress MD virus progression during MDV infection in chickens.

## Discussion

MD is a herpesvirus (MDV)-induced pathology of chickens characterized by paralysis and the rapid appearance of T-cell lymphomas. MDV infection undergoes four stages: early cytolytic infection, latent infection, late lytic infection, and transformation (tumor development and progression). The primary site of MDV latent infection is the activated CD4+ T-lymphocytes in the peripheral blood, resulting in lymphomas in the visceral organs from 3 weeks post-infection (McPherson and Delany, 2016). Accordingly, we collected CD4+ T cells from peripheral blood in this study at 21-days post-infection (late cytolytic phase) for RNA sequencing to investigate whether MDV infection results in transcriptomic level changes of CD4+ T-lymphocytes.

Some research reported that MDV integration into the host genome is involved in tumorigenesis. The *Meq* oncogene is one of the most thoroughly studied MDV genes and is known to play a key role in MDV-induced T cell lymphomagenesis, in combination with other transcription factors, through host and viral gene expression modifications (McPherson and Delany, 2016). As **Figure 6B** showed when CEF cells were co-infected with a negative shRNA and MDV, *Meq* copy numbers slightly decreased compared to that infected with only MDV, which means the negative lentiviral shRNA could interact with *Meq* gene. Therefore, to be normalized, we compared the results of positive shRNAs for the *linc-GALMD1* knockdown with that of a negative shRNA rather than with negative treatments in CEF cells for MDV infection assays. In addition, shRNA4 showed greater *linc-GALMD1* knockdown in CEF cells (**Figure 6A**) while shRNA2 had a greater effect in MSB1 cells (**Figure 2B**), which might be due to MSB1 having integrated copies of the MDV-1 genome that could be affected by shRNA. Thus, for transcriptomic analysis after the *linc-GALMD1* knockdown in MSB1 cells, we analyzed differential expression of genes by comparing samples with a lentiviral shRNA2 infection to samples with an empty lentiviral vector.

From **Figure 6B**, we know that *Meq* copy numbers would significantly increase when *linc-GALMD1* was knocked down, which suggested that *linc-GALMD1* could suppress MDV reproduction during MDV infection in chickens. Furthermore, *linc-GALMD1* expression was higher in non-infected chickens than in infected chickens for resistant line 6_3_, while there was no difference for susceptible line 7_2_ (**Figure 1**), which indicated that *linc-GALMD1* expression would dramatically drop at the late cytolytic stage once line 6_3_ chickens were infected by MDV, but MDV infection did not change *linc-GALMD1* expression for line 7_2_, which implied that *linc-GALMD1* could be a resistant genetic marker of MD or may act synergistically with resistant genes to regulate in tumorigenesis. In our previous study (Han et al., 2017), a lincRNA, names *linc-GALMD3*, was identified highly expressed in MDV-infected CD4+ T cells from F1 generation—two reciprocal cross chicken lines (6_3_ × 7_2_ and 7_2_ × 6_3_), showing that this lincRNA might stimulate the chicken MD. However, *linc-GALMD1* was not identified in F1 generation chickens and its expression was decreased in CD4+ T cells after viral infection in line 6_3_ chickens, implying *linc-GALMD1* might involve suppressing the chicken MD. Recently, our collaborated group also identified lncRNAs in spleen tissue from MDV-infected and non-infected chickens, and revealed informative lncRNA-mRNA and gene-gene interaction networks, hinting that hub genes or lncRNAs exert a critical influence on MD resistance and tumorigenesis (You et al., 2019). Also, some researchers reported that the functions of lncRNAs (e.g., the *ERL lncRNA*) could be disrupted by its hyper-editing during MD viral infections (Figueroa et al., 2016). Therefore, the lincRNA expression is cell/tissue-type specific and its functions are quite diverse that they may act as a virus stimulator or suppressor to directly/indirectly regulate the course of chicken MD.

In resistant chicken genotypes, the latent infection persists at a low level in the spleen and blood lymphocytes without further effect. However, in MD-susceptible chickens, the second wave of cytolytic infection begins around 14–21 dpi after primary infection with a very virulent MDV pathotype. Latently infected peripheral blood lymphocytes disseminate the virus around the body to organs as diverse as the skin, viscera, and nerves (Baigent and Davison, 2004). As **Figure 4B** showed, of 10 DEGs for both resistant line 6_3_ and susceptible line 7_2_, two genes had distinct expression patterns for the two lines. Specifically, the expression of the *IGLL1* gene and 5.8S rRNA gene decreased after MDV infection for line 6_3_, while it increased after MDV infection for line 7_2_. *IGLL1* is immunoglobulin lambda-like polypeptide 1, that encodes one of the surrogate light chain subunits and is a member of the immunoglobulin gene superfamily. *IGLL1* was studied that it is involved in host-virus interaction through copy number variations (CNV) (Budzko et al., 2016). Therefore, *IGLL1* is related to the host’s resistance to the virus and its expression level accompanied phenotypic changes, implying *IGLL1* could be considered as an indicator of MD severity. Serglycin (SRGN) proteins are involved in tumor metastasis and may serve as a mediator of granule-mediated apoptosis (Zhang et al., 2017). Some studies indicated that tripeptidyl peptidase 1 (TPP1) can inhibit tumor growth by interacting with PD-1/PD-L1 (Chang et al., 2015; Li et al., 2018). Our results (**Figure 4C**) showed that once we knocked down *linc-GALMD1* in MSB1 cells, *SRGN* and *TPP1* gene expression were significantly decreased, suggesting that *linc-GALMD1* as a tumor suppressor may inhibit tumor growth *via* coordinating some genes including *SRGN* and *TPP1*. MD viral infection assays demonstrated that *SRGN* and *TPP1* gene expression increased after the MDV infection in resistant line 6_3_ (**Figure 4C**), which implied that *SRGN* and *TPP1* play vital roles in resistance of the birds in response to MDV challenge.

Overall, our studies indicated that *linc-GALMD1* is a viral regulator to suppress tumor progression by repressing expression of MDV genes and regulating immune responses to MDV infection. The interaction of *linc-GALMD1* with MDV represents a novel and sophisticated level of regulation that raises new challenges in terms of its mechanisms of action.

## Materials And Methods

### Animal Challenge Trial and CD4+ T Cell Isolation

Line 6_3_ and line 7_2_, which are known as MD-resistant and MD-susceptible lines, respectively (USDA-ARS, Avian Disease and Oncology Laboratory, East Lansing, Michigan, USA), were used in this study. The animal challenge trials were carried out in accordance with guidelines established and approved by the USDA, ADOL Animal Care and Use Committee (ACUC) (April, 2005), and the Guide for the Care and Use of Laboratory Animals by Institute for Laboratory Animal Research (2011). Chickens from each of the lines/crosses were divided into two groups, one was challenged with a very virulent plus (vv+) strain of MDV (648A passage 40) with a dosage of 500 plaque-forming units (PFU) per bird intra-abdominally in the fifth day post-hatch, the other was maintained as uninfected control. Two chickens were sampled from the MDV challenged groups and two from the control group for each line and each cross. Peripheral blood of the chickens was collected at 21-days post-infection, and peripheral blood mononuclear cells (PBMC) were separated from anti-coagulated peripheral blood by standard preparation method. After removing clumps (if any) and dead cells, PBMC were incubated with Mouse Anti-Chicken CD4-PE (Cat. No. 8210-09, Southern Biotech) at room temperature for 15 min, and then the CD4+ T cells were finally isolated by EasySep^®^ Positive PE Selection Kit (Cat. No. 18557, STEMCELL Technologies) following the manufacturer’s instructions. The cell number and purity were quantified by flow cytometric analysis. Only those cell samples with purity greater than 90% were used for subsequent analyses. Highly pure CD4+ T cell samples were flash-frozen in liquid nitrogen immediately and then stored at −80°C until RNA extraction.

### Transcriptome Sequencing and Analysis in CD4+ T Cells

A total of eight RNA samples were extracted from chicken CD4+ T cells using Direct-zol™ RNA Miniprep Kit (Cat. No. R2050, Zymo Research), and mRNA isolation was performed by Oligotex mRNA Mini Kit (Cat. No. 70022, QIAGEN). Then mRNA was used to synthesize the first and the second strand cDNA using SuperScript™ III Reverse Transcriptase (Cat. No. 18080-093, Invitrogen) and NEBNext^®^ mRNA Second Strand Synthesis Module (Cat. No. E6111S, NEB). After purification, the double-stranded cDNA (dscDNA) was fragmented into ∼300 bp. Then the library for sequencing on the Illumina HiSeq 2000 Analyzer was constructed as previously described (He et al., 2015). Finally, cluster generation and sequencing analysis were performed on the Illumina Hiseq 2000 following the manufacturer’s protocol.

After sequencing quality test by FastQC, the first 6 bp and the last 4 bp for all reads were trimmed off, and then all trimmed reads with 40 bp of length for each sample were mapped to the chicken genome (galGal3) individually by Bowtie 1.2.0. The numbers of reads that fell in each gene were counted by htseq-count, and DEGs between infected and non-infected chickens were analyzed by edgeR with the criteria of |log2fold change| ≥ 1 and FDR ≤ 0.01.

### Long Intergenic Non-Coding Ribonucleic Acid Identification and Analysis

Transcriptomic sequencing reads for each sample were mapped to the chicken genome (galGal3) individually using TopHat 2.0.6 and assembled individually with cufflinks. Putative lincRNAs were identified with our previous pipeline in chickens (He et al., 2015). The expression levels of all protein-coding genes and lincRNAs were estimated using cufflinks and they were represented with FPKM (Fragments Per Kilobase of transcript per Million mapped reads). To obtain the expression patterns of lincRNAs and protein-coding genes, log2 fold changes between infected and non-infected chickens were calculated for each chicken line. Those lincRNAs and protein-coding genes with |log2fold change| ≥ 1 and FDR ≤ 0.1 were selected for substantial analysis. The conservation of non-coding RNAs and enhancers was investigated with the VISTA Enhancer Browser (https://enhancer.lbl.gov).

### Reverse Transcription Quantitative Polymerase Chain Reaction Analysis

The protocols of mRNA extraction and dsDNA synthesis were the same as those mentioned above. Real-time PCR using SYBR Green PCR Kit was utilized to validate differentially expressed lincRNAs and genes in CD4+ T cells between infected and non-infected chickens with iCycler iQ PCR System (Bio-Rad). The annealing temperature was set at 60°C. The primer pairs were designed within exons and the length of the amplicons was between 50 and 200 bp. All primers were designed using Primer3 (http://fokker.wi.mit.edu/primer3/input.htm), and the detailed information is listed in **Table S1**. qPCR reaction was run with triplicate using the program as follows: pre-incubation (95°C for 10 min), 40 cycles of amplification (95°C for 10 s, 60°C for 10 s, and 72°C for 10 s), melting curves using a heat ramp and cool down. Cycle threshold values (Ct values) were obtained from iCycler iQ PCR software. The expression levels of lincRNAs and genes were normalized against *GAPDH* complementary DNA in the corresponding samples. The relative fold enrichment of each treatment group was calculated by comparing the enrichment value for the given primer pair to *GAPDH*. The copy numbers of the *Meq* gene were calculated against *PCCA* as the reference gene (Wang et al., 2010). Data were analyzed using a two-tailed Student’s t-test. The differences were considered to be statistically significant at p-value < 0.05.

### Structure Validation of *linc-GALMD1*

To confirm the structure of *linc-GALMD1* in CD4+ T cells and MSB1 cells, the primer pairs were designed to span over two exons of *linc-GALMD1* and the length of the amplicons was between 80 and 500 bp (**Table S2**). Touchdown-PCR was performed to amplify *linc-GALMD1* with conventional PCR, and chicken genomic DNA was used as control using the following reaction: 5 µl of GoTaq^®^ Hot Start Green Master Mix (Promega, USA), 1 µl of primer mix (10 µM), 1 µl of cDNA, and 3 µl of UltraPure^®^ Distilled Water (Invitrogen, USA) to a total volume of 10 µl. The optimal PCR program was 94°C for 5 min, 3 cycles of amplification (94°C for 1 min, 68°C for 1 min, and 72°C for 2 min), 3 cycles of amplification [94°C for 1 min, (68-3i)°C (i = 1 to 5) for 1 min, and 72°C for 2 min], 30 cycles of amplification (94°C for 1 min, 50°C for 1 min, and 72°C for 2 min), and a final extension step 72°C for 10 min. PCR products were run on 1.5% TBE-buffered agarose gel at 90 V for 1 h, and 230 bp fragments were excised from the gel to purify by QIAquick Gel Extraction Kit (QIAGEN, USA).

To confirm that sequences of *linc-GALMD1* existed in CD4+ T cells and MSB1 cells, cloning sequencing of PCR products was conducted. The details were as follows. The purified PCR products were ligated into pGEM-T Vector (pGEM-T Vector System I, Promega, USA), transformed into DH5α competent cells (Z-Competent E. Coli Cells—Strain Zymo 5α, ZYMO Research, USA), and screened for successful insertions (blue-white selection) after incubation at 37°C overnight. In the next step, five white colonies from each sample were cultured overnight in a 37°C shaker. Plasmid DNA was isolated using Zyppy Plasmid Miniprep Kit (ZYMO Research, USA). M13 reverse primer and BigDye Terminator v3.1 Cycle Sequencing Kit (Applied Biosystems, USA) was employed for sequencing in the ABI 3730 machine as described by the manufacturer.

### *Linc-GALMD1* Knockdown by Short Hairpin Ribonucleic Acids

The MDV-transformed lymphoblastoid MSB-1 cells were obtained from Dr. C. Itakura’s lab, Department of Veterinary Pathology, Tottori University, Tottora, Japan and grown at 37°C in 5% CO_2_ in RPMI 1640 medium containing 10% fetal bovine serum. The chicken embryo fibroblast (CEF) cells were isolated from fertile eggs at day 10 and grown at 37°C in 5% CO_2_ in M199 tissue culture medium supplemented with 10% FBS. All reagents for cell culture were purchased from Life Technologies (CA, USA).

The shRNAs were designed and hypothesized by GenePharma Biotech (Shanghai, China). Four shRNAs for interfering *linc-GALMD1* and a negative control (NC) were cloned into a shuttle vector (LV3-pGLV-h1-GFP-puro) labeled by GFP (green fluorescent protein) and incorporated into a lentiviral vector, respectively. The sequences of shRNAs were listed in **Table S3**.

MDCC-MSB1 cells were plated at a density of 5×10^5^ cells per well in 24-well plates. Cells were infected with 40 µl of a lentiviral shRNA stock (2×10^8^ TU/ml) and incubated at 37°C for 72 h until green fluorescence was observed by fluorescence microscope. The total RNA extraction and qRT-PCR were performed as described above.

CEF cells were plated at a density of 4×10^5^ cells per well in 24-well plates. They were co-infected with 2000 PFU MDV (CVI988, Beijing Lingyu Biological Technology Co., Ltd.) and 40 µl of lentiviral shRNA2 or shRNA4 plus NC (2×10^8^ TU/ml). The cell lesion and green fluorescence were observed at 96 h after infection, and then cells were harvested for isolating the total RNAs and DNA.

### Transcriptome Sequencing and Analysis in MSB1 Cells

MDCC-MSB1 cells were plated in 24-well plates, and cells were infected with a lentiviral shRNA2 and an empty lentiviral vector as well as a NC in triplicates. After cells were harvested, the total RNAs were extracted and dscDNA was produced as described above. Then the library for sequencing on the Illumina HiSeq 2500 Platform was generated using the NEBNext^®^ Ultra^™^ Directional RNA Library Prep Kit for Illumina^®^ (Cat. No. E7420L, NEB) following the manufacturer’s recommendations and index codes were added to attribute sequences to each sample. Products were purified by QIAquick PCR Purification Kit (Cat No. 28106, QIAGEN), and library quality was assessed on Qubit^®^ Fluorometer by Qubit^®^ dsDNA HS Assay Kit (Cat. No. Q32851, Thermo Fisher). The clustering of the index-coded samples was performed on a cBot Cluster Generation System using TruSeq PE Cluster Kit v3-cBot-HS (Cat. No. PE-401-3001, Illumina) following the manufacturer’s protocol. After cluster generation, the library preparations were sequenced on an Illumina HiSeq 2000 V4 PE125 platform and paired-end reads were generated.

After filtering out paired reads with adaptors and dirty reads, all clean reads for each sample were mapped to the chicken genome (galGal4) individually by Tophat v2.0.9, and the average concordant pair alignment rate was 70%. Mapped transcripts were assembled individually with cufflinks and then transcripts from all samples were merged together with cuffmerge to build a consensus set of transcripts across samples. DEGs between samples with *linc-GALMD1* knockdown and with negative lentiviral vector were screened by cuffdiff based on cuffmerge results. Gene expression levels were represented with FPKM (Fragments Per Kilobase of transcript per Million mapped reads) and log2 transformation was used for normalization. To obtain gene expression patterns, log2 fold change before and after the *linc-GALMD1* knockdown in MSB1 cells was calculated. Those genes with |log2fold change| ≥ 1 and FDR ≤ 0.01 were selected for clustering of expression profiles based on hierarchal clustering with complete linkage and visualized using heatmaps.

## Data Availability Statement

Sequencing data can be retrieved from the links of https://www.ebi.ac.uk/ena/data/view/PRJEB35191.

## Ethics Statement

USDA, ADOL Animal Care and Use Committee (ACUC) (April, 2005) guidelines and the Guide for the Care and Use of Laboratory Animals were established by Institute for Laboratory Animal Research (2011).

## Author Contributions

JS and NY conceived and designed this experiment. HZ and SC conducted the MDV challenge trials in chickens. YH and YD collected the samples and isolated the CD4+ T cells. YH implemented all bioinformatics analysis. BH performed the cell culture, lincRNA knockdown, MDV infection assays, and RT-qPCR experiments, with the help of LZ and CZ. YH wrote the manuscript. All authors read, revised, and approved the final version of the manuscript.

## Conflict of Interest

The authors declare that the research was conducted in the absence of any commercial or financial relationships that could be construed as a potential conflict of interest.

## Supplementary Material

Supplementary Table 1 Primers used to confirm lincRNA expression by qPCR.

Supplementary Table 2 Primers used to confirm linc-GALMD1 structure.

Supplementary Table 3 shRNA sequences for linc-GALMD1 interference.

Supplementary Figure 1 The validation of differentially expressed lincRNAs by qPCR. Dashed line: the threshold line corresponds to the ratio of 1. When the ratio is more than 1, lincRNA expressed more in CD4+ T cells of infected chickens than in non-infected chickens, and when the ratio is less than 1, lincRNA expressed less in CD4+ T cells of infected chickens than in non-infected chickens.

Supplementary Figure 2 The conservation of *linc-GALMD1* and neighboring enhancers across the chicken, human, and mouse. The upper panel is the differential expression of twenty neighboring genes of *linc-GALMD1* as in **Figure 3B**. The lower panel is the conservation of *linc-GALMD1* and two neighboring enhancers across the three species. The downstream enhancer was found to be close to *AKTIP* gene and it is conservative across the chicken (chr11: 5,492,800-5,494,599, galGal3), human (chr16: 53,578,034-53,578,985, hg19), and mouse (chr8: 93,709,198-93,710,130, mm9).

Supplementary Figure 3 The heatmap of differentially expressed genes in CD4+ T cells between infected and non-infected chickens with two replicates in line 6_3_ or 7_2_ (|log2Fold change| ≥ 1 and FDR ≤ 0.1). Red to yellow: gene expression from high to low.

Supplementary Figure 4 Gene Ontology Annotation Plotting of differentially expressed genes between infected and non-infected chickens for lines 6_3_ and 7_2_ by WEGO.
