# Supplementary material for: Linc-GALMD1 Regulates Viral Gene Expression in the Chicken
Source: Front Genet. 2019 Nov 14;10:1122. doi: 10.3389/fgene.2019.01122 (PMC6868033; doi:10.3389/fgene.2019.01122)
Supplement: Supplementary Table 1 — Primers used to confirm lincRNA expression by qPCR. [file DataSheet_1.docx]

###### **SUPPLEMENTARY TABLE 1 |** Primers used to confirm lincRNA expression by qPCR

| **qPCR** | **Primer name** | **Sequence (5’ → 3’)** | **Product Length (bp)** | **Template** | **Annealing temperature (°C)** |
| --- | --- | --- | --- | --- | --- |
| Linc-GALMD1 | 438 | F: 5’ GGCTGCTGGGAAAGAGAAGT 3’ | 54 | dscDNA | 60 |
|  |  | R: 5’ GTCACCGTACCTCACTGAGC 3’ |  |  |  |
| lincRNA2 | 950 | F: 5’ GGGGAAATGTGGAGAAGGCA 3’ | 54 | dscDNA | 60 |
|  |  | R: 5’ CCCCTTTTCCCTTCAGCCTT 3’ |  |  |  |
| lincRNA3 | 035 | F: 5' AGCTTTGTTCTGGTCTCGCA 3' | 82 | dscDNA | 60 |
|  |  | R: 5' TGTCCGTTCCCATTACAGCC 3' |  |  |  |
| lincRNA4 | 595 | F: 5' CATGAACCCACCGCCTGAA 3' | 69 | dscDNA | 60 |
|  |  | R: 5' CTTCCAGGCTCGGCTCAC 3' |  |  |  |
| lincRNA5 | F748 | F: 5' TGCTCTCTCTCCTTTGCTGC 3' | 100 | dscDNA | 60 |
|  |  | R: 5' AGGATTGTCTCGCTGCAACA 3' |  |  |  |
| lincRNA6 | F71 | F: 5' TCCCTGTTTGAGCTGCTCTG 3' | 111 | dscDNA | 60 |
|  |  | R: 5' AACTCTGCCTCACAACTCGG 3' |  |  |  |
| lincRNA7 | F72 | F: 5' AGCCTCTGAATGGCACTTCC 3' | 131 | dscDNA | 60 |
|  |  | R: 5' GACACATGGGCTGACAGTGA 3' |  |  |  |
| lincRNA8 | F26 | F: 5' AGTCAGGGCATGTGTCTTGG 3' | 74 | dscDNA | 60 |
|  |  | R: 5' CAGGAACAACGTGCATGCAA 3' |  |  |  |
| lincRNA9 | F01 | F: 5' CTTTCCGGGCACAGTGTTTG 3' | 97 | dscDNA | 60 |
|  |  | R: 5' ACTACTTGAGGGTTGGCACG 3' |  |  |  |
| lincRNA10 | F05 | F: 5' GGGTGGGAAGGAAAGGATGG 3' | 54 | dscDNA | 60 |
|  |  | R: 5' CCCCTTTGGCTGTTTGTTCG 3' |  |  |  |
| lincRNA11 | F48 | F: 5' TCTGCCAACTGAGTGCTAGC 3' | 82 | dscDNA | 60 |
|  |  | R: 5' GCAGTTGTGGAGGACGGTTA 3' |  |  |  |
| lincRNA12 | F44 | F: 5' AGATGACAGGGGCAATGGTG 3' | 111 | dscDNA | 60 |
|  |  | R: 5' GGAAAGGATGGGAAGGCACA 3' |  |  |  |
| lincRNA13 | F83 | F: 5' CCCACGTAGCATCCAAAGGT 3' | 153 | dscDNA | 60 |
|  |  | R: 5' CTGTTAGGAGCTGTGGTGGG 3' |  |  |  |
| lincRNA14 | F64 | F: 5' ACTCCTCCTGCATGAACACG 3' | 55 | dscDNA | 60 |
|  |  | R: 5' GTGGGAACTCGGCACCTTTA 3' |  |  |  |
| lincRNA15 | F17 | F: 5' AAGGCAGCAAGTGGTCTACC 3' | 59 | dscDNA | 60 |
|  |  | R: 5' CGCTGCACTGGAGTTAATGC 3' |  |  |  |
| lincRNA16 | F71 | F: 5' TCCCTGTTTGAGCTGCTCTG 3' | 111 | dscDNA | 60 |
|  |  | R: 5' AACTCTGCCTCACAACTCGG 3' |  |  |  |
| GAPDH | HK-1 | F: 5' GAGGGTAGTGAAGGCTGCTG 3' | 307 | dscDNA | 60 |
|  |  | R: 5' ACCAGGAAACAAGCTTGACG 3' |  |  |  |

###### **SUPPLEMENTARY TABLE 2 |** Primers used to confirm linc-GALMD1 structure

| **PCR** | **Primer name** | **Sequence (5’ → 3’)** | **Product Length (bp)** | **Template** | **Annealing temperature (°C)** |
| --- | --- | --- | --- | --- | --- |
| Linc-GALMD1 | Stru-Primer1-1 | F: 5’ AGCTGTAGATTGTCACCTGTTG 3’  R: 5’ GAGTCCACCAAGAAAACAGGA 3’ | 230 | dscDNA | 55 |

###### **SUPPLEMENTARY TABLE 3 |** shRNA sequences for linc-GALMD1 interference

| **shRNA** | **Sequence** |
| --- | --- |
| shRNA1 | GCCACTCCATAGCATGGATTC |
| shRNA2 | GGCATTACTCATGAACAAAGC |
| shRNA3 | GGCAGGACAAATTCATAAAGC |
| shRNA4 | GGATTGACTTGCTGCTGATTT |
| LV3 (Negative Control) | TTCTCCGAACGTGTCACGTTTC |

**The sequence of linc-GALMD1:**

**TCONS_00004562**

>galGal3_ct_UserTrack_3545_TCONS_00004562_0 range= chr11:3450045-3450142 5'pad=0 3'pad=0 strand=+ repeatMasking=none

AATTCTGTGTTCACTTTCTCTCAGGATCACTGTAGGCAAATCATGCCAGAAGCTGTAGATTGTCACCTGTTGGGGATAGAAAAGGACACCTTGCACTT

>galGal3_ct_UserTrack_3545_TCONS_00004562_1 range= chr11:3451011-3452326 5'pad=0 3'pad=0 strand=+ repeatMasking=none

GACAGCTTACTTAGACCAGATGTTTTTAGTTAAGAGTTTTATACAGTCAGAAACAGGAACGAAGACAGAAGACTAAGCATAAACTAAGTCACTCAACAGTAAGTTCTCATCTTGCCCTTGTATATAAAATTATTTGTCCCCTGTCAATTAATCATTAATTTTCCTGTTTTCTTGGTGGACTCTAGACTCTTATACTTGTGAGCAGTAATCAAATGGAATTCAACCTTAAGAGGTTACAGAAAATGCTACCTGTTGGTCATTTTTTTTCTGCATTTCTCTGGGGGAAAATACACTGCTGGAGATAACTGAGTGGATGGAGTAGCACACCAGCATGCTTCCATGTCAGTACTAACATTTGTAAAATAACAAAAAATAACAAAAAAAAAAAAACCTTTCTAAATTACCCACCCACAGAAGTTGTTAGTAAACTTCACAGACTTCAGTGTGATTGTATACAAAAACGTGCTAATGTTAGGGCTGCTGGGAAAGAGAAGTGATCTCACTCTCTAGCTCAGTGAGGTACGGTGACTTTTGAATGGAAGCTGGTTTTGTTTCTTCCCAGATCTTCTGAGGCTGTTACTGAGATACACACGGTCACATGAAGATACACTGTTGTCATCCTTAAGTGAGCAAAATGGCACAAGTAAAATGCAGTGCGTATGGAAACACAGGAGTCTTAGAAGTCTTTCCGCTTGTCTGATCAGTGCTAGGAGGTGTAATTGTTTTGGCTTTCCCCAGAAGTGGTCTATCGACAAGTTTGTGTTAAGTGAACTGAGAGACCTGTAACCCCTTGGCTGCAGCCACTCCATAGCATGGATTCTGACCGTTTATTTCAAAACCCACCTAATTAAGAATAGGCATTACTCATGAACAAAGCATTTTCCCTCTTGCTTGTCTCTCATCTGCTCATTACAGGTTTTTCTTTCTCCAAGTGACCTAGGGGTTGTTTAGTCAAAAAGGCAGGACAAATTCATAAAGCTAGAGATAAGGTTCTTCTTAGTACAAGGAATGTTGAAAGCGTCTTCCCTCTGTCTCTGCTCTAGGGCTTCTATCCTCCCATAGACTCCTGTATAGAACAGAACTTACAATAGTGCTGGATTGACTTGCTGCTGATTTATTAAGATGTTTTGTAGATTTTTGATTAAAAATACTAGTTCGAGTTGACACTGTAGTAAAACACATGTAGCAAAATGTACAAATGGTTCTGTAAAGCTTTCTCCTCTGATGACTGTGTAGTATATTAAATTGGGATCCTTTTACTTTCAAGAGTAGCTTTCAAAAACAGACAATAAATAGCATTAGCAACTATTAATTGA
