# Supplementary figures and images for: Linc-GALMD1 Regulates Viral Gene Expression in the Chicken
Source: Front Genet. 2019 Nov 14;10:1122. doi: 10.3389/fgene.2019.01122 (PMC6868033; doi:10.3389/fgene.2019.01122)

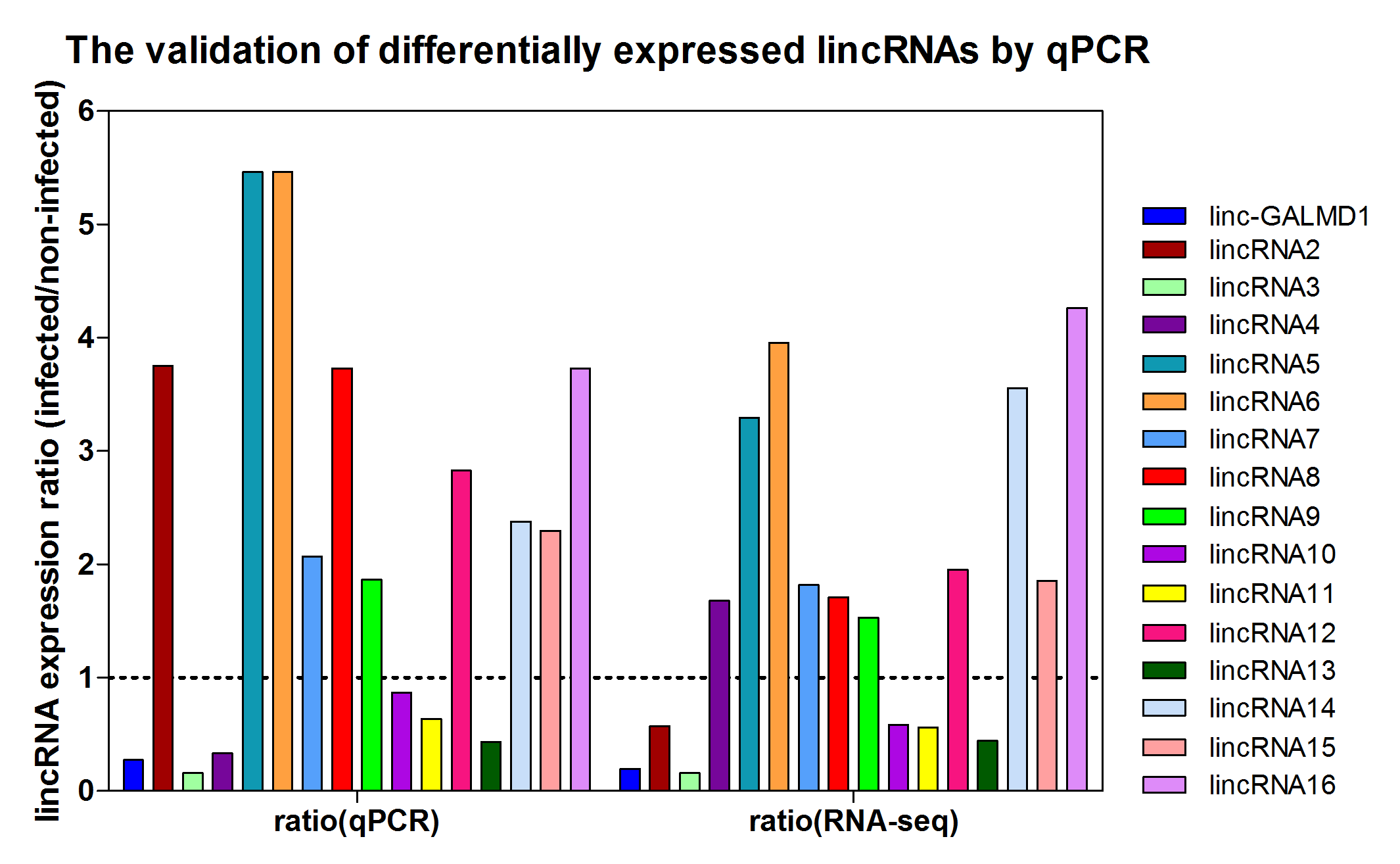

Supplement: Supplementary Figure 1 — The validation of differentially expressed lincRNAs by qPCR. Dashed line: the threshold line corresponds to the ratio of 1. When the ratio is more than 1, lincRNA expressed more in CD4+ T cells of infected chickens than in non-infected chickens, and when the ratio is less than 1, lincRNA expressed less in CD4+ T cells of infected chickens than in non-infected chickens. [file Image_1.tif]

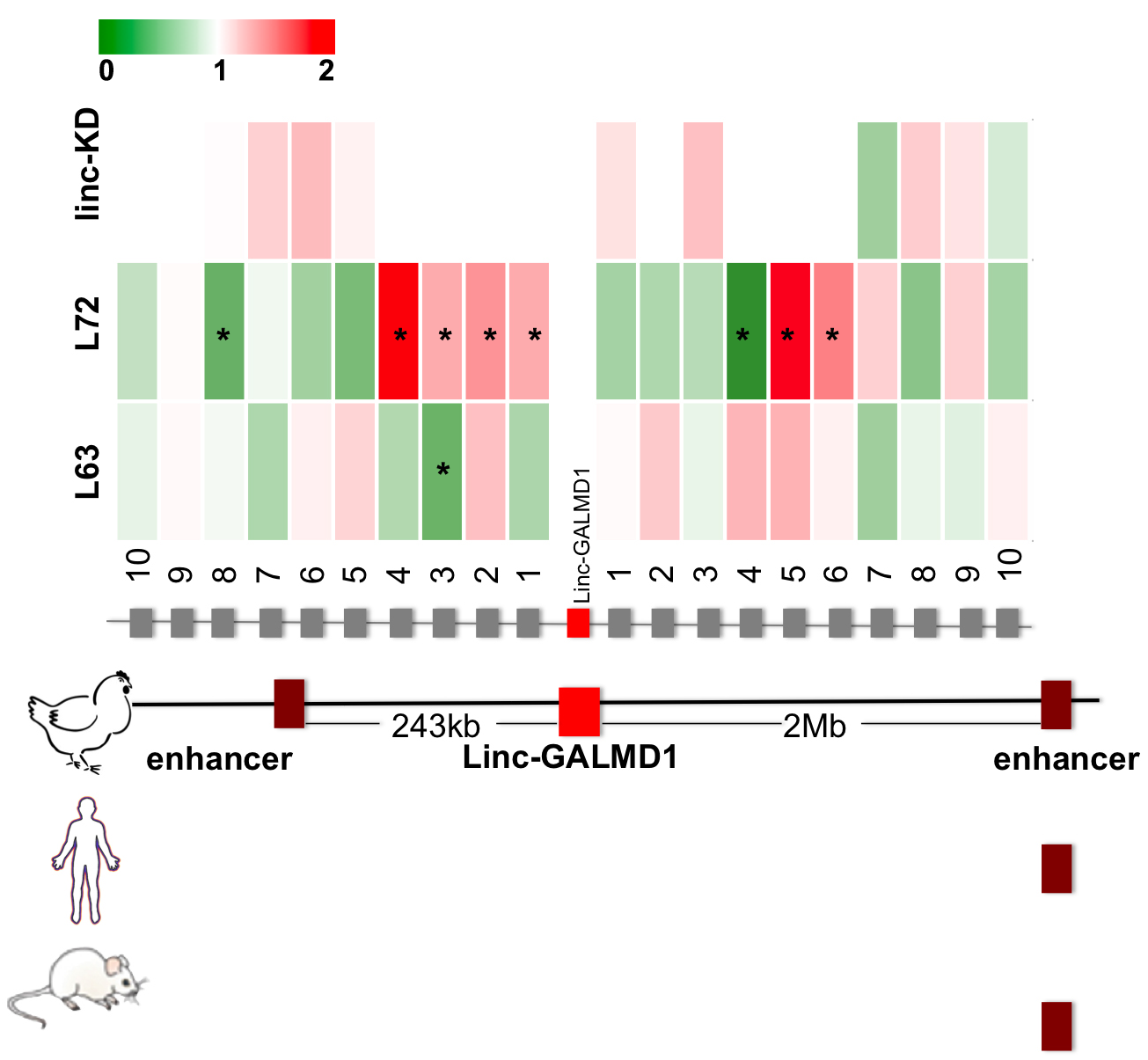

Supplement: Supplementary Figure 2 — The conservation of linc-GALMD1 and neighboring enhancers across the chicken, human, and mouse. The upper panel is the differential expression of twenty neighboring genes of linc-GALMD1 as in Figure 3B . The lower panel is the conservation of linc-GALMD1 and two neighboring enhancers across the three species. The downstream enhancer was found to be close to AKTIP gene and it is conservative across the chicken (chr11: 5,492,800-5,494,599, galGal3), human (chr16: 53,578,034-53,578,985, hg19), and mouse (chr8: 93,709,198-93,710,130, mm9). [file Image_2.jpeg]

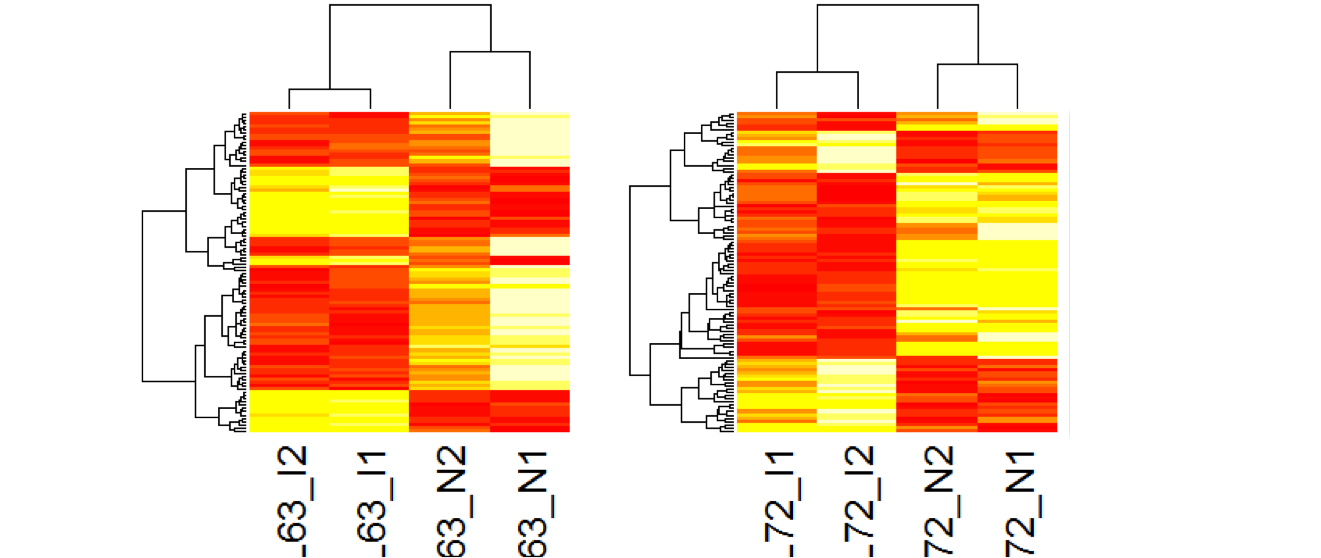

Supplement: Supplementary Figure 3 — The heatmap of differentially expressed genes in CD4+ T cells between infected and non-infected chickens with two replicates in line 63 or 72 (|log2Fold change| ≥ 1 and FDR ≤ 0.1). Red to yellow: gene expression from high to low. [file Image_3.png]

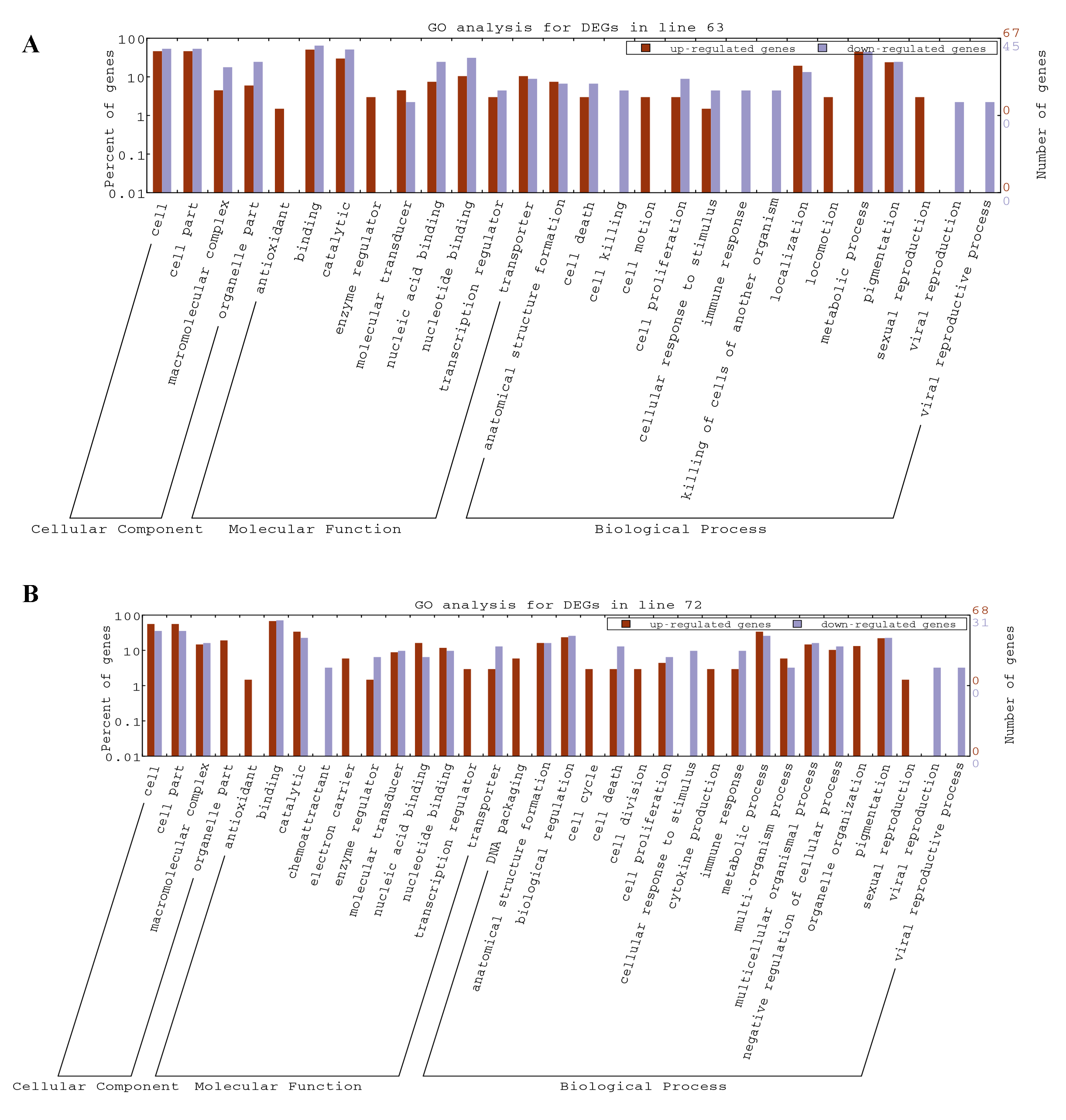

Supplement: Supplementary Figure 4 — Gene Ontology Annotation Plotting of differentially expressed genes between infected and non-infected chickens for lines 63 and 72 by WEGO. [file Image_4.tif]
